# Police and clinician diversion of people in mental health crisis from the Emergency Department: a trend analysis and cross comparison study

**DOI:** 10.1186/s12873-015-0040-7

**Published:** 2015-07-10

**Authors:** Brian McKenna, Trentham Furness, Steve Brown, Mark Tacey, Andrew Hiam, Morgan Wise

**Affiliations:** School of Nursing, Midwifery and Paramedicine, Australian Catholic University, 115 Victoria Parade, 3065 Fitzroy, Australia; NorthWestern Mental Health, Level 1 North, City Campus, The Royal Melbourne Hospital, Grattan Street, 3050 Parkville, Victoria Australia; Northern Area Mental Health Service, NorthWestern Mental Health, The Northern Hospital, 185 Cooper Street, 3076 Epping, Australia; Melbourne EpiCentre, The Royal Melbourne Hospital and Department of Medicine, The University of Melbourne, Grattan Street, 3050 Parkville, Australia; Epping Police Station, Victoria Police, Police Station, 785 High Street, 3076 Epping, Australia

**Keywords:** Emergency department, Police, Mental health, Crisis management

## Abstract

**Background:**

The Northern Police and Clinician Emergency Response (NPACER), a combined police and clinician second response team, was created to divert people in mental health crisis away from the hospital emergency department (ED) to care in the community or direct admission to acute inpatient services. The aim of this study was to evaluate the NPACER by comparing trends in service utilisation prior to and following its inception.

**Methods:**

A retrospective comparison of electronic records was undertaken with interrupted time series analysis to assess the impact of NPACER on ED presentations over 27-months (*N* = 1776). Chi-squared tests were used to analyze service utilization; (1) in the six-months before and after the implementation of NPACER and (2) within the post NPACER period between times of the day it was operational.

**Results:**

NPACER reduced the number of mental health crisis presentations to the ED. When the NPACER team was operational, 16 % of people in crisis went to ED compared with 100 % for all other times of the day, over a six-month period. The NPACER team enabled direct access to the inpatient unit for 51 people assessed at a police station and in the community compared with no direct access when NPACER was not operational.

**Conclusions:**

NPACER enabled reductions in presentations to the ED by diverting people to more appropriate and less restrictive environments. The model also facilitated direct admission to acute inpatient mental health services when people in crisis were assessed in the community or transported to a police station for assessment.

## Background

An increasing number of presentations to emergency departments (EDs) is systemic to hospitals across Australia [1, 2]. Approximately 4 % (*n* = ~243,000) of all ED presentations are mental health related [3] and require admission to an inpatient unit more frequently than all other modes of presentation [4]. Across five metropolitan EDs in the state of Victoria, 24 % of mental health related presentations required psychiatric inpatient admission [5]. However, access to specialist mental health assessment in the ED may be delayed by long ED waiting periods [6, 7], confusion about triage priority [8], potential for violence and aggression [9], subsequent security calls [10], and the use of restrictive interventions [11].

In the state of Victoria, police officers are lawfully entitled under mental health legislation (section 10, Mental Health Act 1986) to detain people in community based mental health crisis, if deemed at risk to themselves or others, and transport them to an appropriate location for specialist mental health assessment [10, 12]. As such, police escorted section 10 ED presentations are common [13]. Of all mental health crisis arrivals to five different metropolitan Victorian EDs, presentation with police officers ranged between 14 and 26 % [10].

In 2012, at one Victorian metropolitan Area Mental Health Service (AMHS), the Northern Police and Clinician Emergency Response (NPACER) team was created to divert the number of section 10 presentations of people in mental health crisis away from the ED to care in the community via referral to appropriate community agencies or diversion directly to acute inpatient service for the acutely unwell. This development is intended to enable police officers who have responded to an emergency call-out to initiate the NPACER specialist second responder team. The NPACER team comprises of a police officer and a senior mental health clinician, usually a mental health nurse. The goal of the joint response is to reduce the potential for violence and provide alternate care to EDs in less restrictive environments through interagency collaboration [14]. The NPACER team only becomes clinically involved once the initial incident has been resolved on a ‘safety first’ principle by first responder police. In situations where safety may be compromised (e.g., siege) the NPACER team may only be used to provide antecedent psychiatric information to first responder police.

Given that processing through EDs is a major concern for people in mental health crisis and their carers [5, 8], processes that allow ED diversion are valuable. However, there is limited evaluation of the ability of second responder units to divert people in acute mental health crisis to a less restrictive alternative for assessment and treatment. Lamb et al. [14] described the success of a police and clinician second responder team in diverting people in crisis with a history of violence away from custody to less restrictive alternatives (e.g., hospital). Therefore, the aim of this present study was to evaluate the NPACER team by comparing trends in service utilisation of people in mental health crisis prior to and following its inception.

## Methods

A retrospective comparison of electronic records was undertaken at the Northern Hospital, Northern Health, to determine service utilisation for all people in mental health crisis with a legal status of section 10. This study was approved by the Melbourne Health Office for Research (QA2013128).

### NPACER

**Fig. 1:**
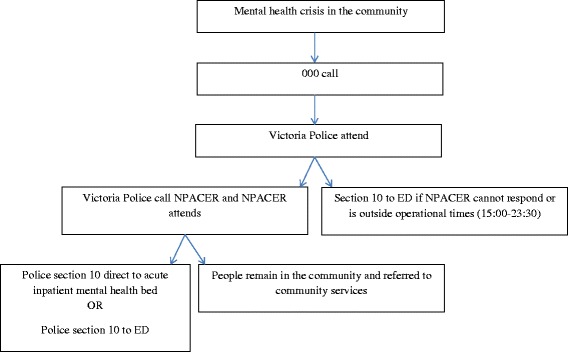
Operational flow of the NPACER model

The NPACER team is collaboratively supported by NorthWestern Mental Health, Melbourne Health, Northern Health, and Victoria Police. A senior mental health clinician and a member of Victoria Police act as second response to acute mental health crisis in the community. Clinicians are drawn from a limited pool of senior emergency mental health nurses in the service, while the police officers are drawn from a wider pool of rostered staff, cognisant of experience and support for the NPACER initiative. The NPACER unit attends call-outs in a marked Victoria Police vehicle and allows mental health assessment *in situ* (Fig. 1). Based on the perceived need for section 10 events, the NPACER team operates seven days a week, every afternoon/evening (15:00–23:30 h), across two Victoria Police Divisions growth corridors (approximately 600,000 people) [15] characterised with low socio-economic status, high immigrant, and ethnic diversity compared with the remainder of the state of Victoria [16]. To assist with ED diversion for the acutely unwell requiring hospitalisation, three inpatient beds are made available to NPACER to allow direct admission.

### Data collection

The frequencies of section 10 use within Northern AMHS were collected across a 27-month period (November 2011 to January 2014). A detailed analysis of service utilisation was undertaken comparing the six-months prior to the implementation of NPACER (May to October 2012) and the six-months following (February to July 2013). Electronic records were accessed for all people in mental health crises placed under section 10 to describe service utilisation including access to acute mental health inpatient services; (1) prior to NPACER and (2) following implementation of NPACER. During the NPACER period, data were dichotomised by; (1) when NPACER was operational (i.e., 15:00–23:30 h) and (2) all other times of the day. This enabled a second comparison between time of the day when the NPACER was operational and time of the day when it was not.

### Data analysis

Interrupted time series analysis was conducted using simple linear regression to assess the impact of NPACER on the number of ED presentations over the 27-month period. Descriptive statistics and Chi-squared tests were used to present the changes in categorical variables; (1) in the period prior to NPACER with the period following the implementation of NPACER and (2) within the NPACER period between time of the day it was operational and all other times of the day. All statistical tests were two-sided and conducted at a significance level of α = 0.05. Statistical analysis was performed using Stata version 12.1 (Stata Corp LP, Texas, USA).

## Results

### Section 10 trends; November 2011 to January 2014

**Fig. 2:**
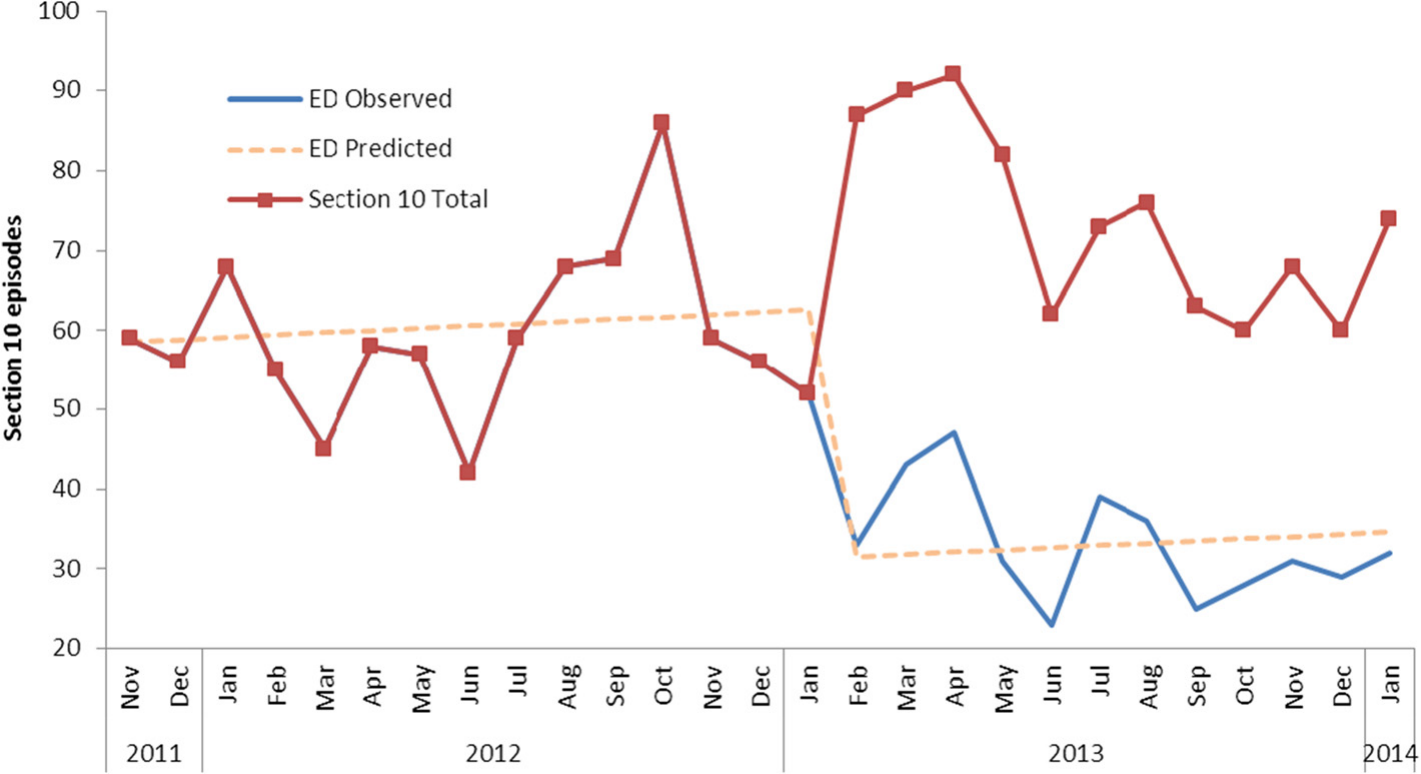
Effects of NPACER on section 10 presentations to the ED. NPACER commenced in November 2012. Trend analysis excludes a three month embedding process (November 2012 – January 2013)

A total of 1776 section 10 episodes occurred in the mental health service from November 2011 to January 2014 (Fig. 2). The introduction of NPACER coincided with an approximate 50 % reduction (*p* = 0.04) in the number of section 10 arrivals to the ED, from a mean of 60.1 per month (November 2011 to October 2012) to 33.1 per month (February 2013 to January 2014). The NPACER team attended 490 (55 %) of 887 call-outs from February 2013 to January 2014.

### Pre compared with Post NPACER period

A total of 796 section 10 episodes occurred at the mental health service for the six-months pre (May to October 2012) and post NPACER implementation (February to July 2013) (see Table 1). During the post NPACER period, there was a reduction in the frequency of people in mental health crisis who were without a primary diagnosis (*p* < 0.01). All 359 people on section 10 in the pre NPACER were assessed at the ED compared with 234 (54 %) in the post NPACER period (*p* < 0.01). In the post NPACER period there were 70 assessments at police stations and 133 in the community. A larger frequency of people in crisis was discharged to the community during the post NPACER period (65 %) compared with the pre NPACER period (51 %) (*p* < 0.01).

**Table 1:** Descriptive variables of people under section 10 in the six-month periods before and after the introduction of NPACER

Variable		May – Oct 2012 (*N* = 359)	Feb – July 2013 (*N* = 437)	*p* value
Gender (%)				0.28
	Female	134 (37)	171 (39)	
	Male	225 (63)	266 (61)	
Age (years)	Mean (SD)	33.2 (12.2)	34.4 (12.3)	0.17
Primary Diagnosis (%)				0.02*
	No diagnosis^a^	75 (20.9)	50 (11.4)	
	Situational crisis^b^	69 (19.2)	100 (22.9)	
	Psychotic illness^c^	74 (20.6)	78 (17.9)	
	Affective disorders^d^	59 (16.4)	85 (19.5)	
	Anxiety disorder^e^	8 (2.2)	6 (1.4)	
	Alcohol/drugs^f^	7 (1.9)	33 (7.6)	
	Intellectual disability^g^	4 (1.1)	4 (0.9)	
	Personality disorder^h^	63 (17.6)	81 (18.5)	
Initial pathway for assessment (%)				0.01
	ED	359 (100)	234 (54)	
	Community^i^	0 (0)	133 (30)	
	Police station	0 (0)	70 (16)	
	Disposition^j^ (%)			0.01
	Community	179 (51.1)	281 (65.4)	
	Police custody	25 (7.1)	12 (2.8)	
	Mental health unit	141 (40.3)	133 (30.9)	
	Missing	5 (1.4)	4 (0.9)	

*p-value excludes those with no diagnosis

^a^No diagnosis – mental health assessment yet to be completed

^b^Situational crisis – acute stress reaction, suicidal ideation/threat, post-traumatic stress disorder

^c^Psychotic illness – schizophrenia spectrum disorders, drug induced psychosis

^d^Affective disorders – depression, bipolar affective disorder

^e^Anxiety disorder – social phobias, ruminations, obsessive compulsive disorder

^f^Alcohol/drugs – ethanol, cannabis, methamphetamines

^g^Intellectual disability – borderline intellectual functioning, learning disability, Aspergers Syndrome

^h^Personality Disorder – schizo-atypical, schizoid, anti-social, borderline, dependent, conduct disorder

^i^Community – home, public place, general practitioner, psychologist, community mental health service, supported accommodation

^j^Where the person in crisis went after the section 10 event

**Table 2:** Socio-demographic characteristics and diagnosis of people under section 10 within the six-month period after the introduction of NPACER

Variable		Non-NPACER (*N* = 194)	NPACER (*N* = 243)	*p* value
Gender (%)				0.75
	Female	78 (40.2)	101 (41.7)	
	Male	116 (59.8)	141 (58.3)	
Age (years)	Mean (SD)	34.2 (11.8)	34.6 (12.8)	0.88
Primary Diagnosis (%)				0.58
	No diagnosis^a^	23 (11.9)	27 (11.1)	
	Situational crisis^b^	45 (23.2)	55 (22.6)	
	Psychotic illness^c^	34 (17.5)	44 (18.1)	
	Affective disorders^d^	40 (20.6)	45 (18.5)	
	Personality disorder^e^	29 (14.9)	52 (21.4)	
	Anxiety disorder	4 (2.1)	2 (0.8)	
	Alcohol/drugs^f^	17 (8.8)	16 (6.6)	
	Intellectual disability^g^	2 (1.0)	2 (0.8)	

^a^No diagnosis – mental health assessment yet to be completed

^b^Situational crisis – acute stress reaction, suicidal ideation/threat, post-traumatic stress disorder

^c^Psychotic illness – schizophrenia spectrum disorders, drug induced psychosis

^d^Affective disorders – depression, bipolar affective disorder, social phobias, ruminations, obsessive compulsive disorder

^e^Personality Disorder – schizo-atypical, schizoid, anti-social, borderline, dependent, conduct disorder

^f^Alcohol/drugs – ethanol, cannabis, methamphetamines

^g^Intellectual disability – borderline intellectual functioning, learning disability, Asperger

### Within the Post NPACER period

A total of 437 section 10 episodes occurred at the mental health service within the six-months of the NPACER period (see Table 2). There was no difference among primary diagnosis between when the NPACER was operational and when not (*p* = 0.58). For both, most people in mental health crisis were afflicted with situational crisis and the frequency of people with no diagnosis was similar (12 % non-NPACER, 11 % NPACER).

**Fig. 3:**
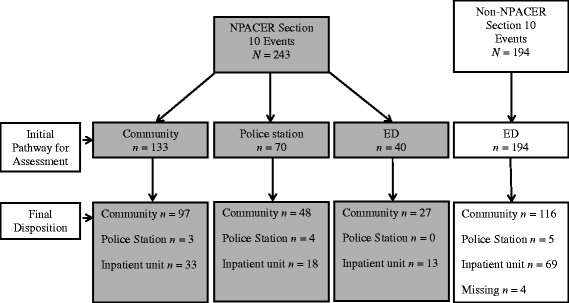
The pathway of section 10 events with NPACER and without NPACER

All 194 people on section 10 in the non-NPACER period were assessed at the ED compared with 40 (16 %) in the NPACER period (*p* < 0.01) (see Fig. 3). The NPACER allowed direct access to the inpatient unit for 51 people when assessed at a police station and the community. Sixty-nine non-NPACER people in crisis were admitted to the inpatient unit after processing through the ED. Of the 133 people NPACER assessed in the community, 73 % (*n* = 97) remained at that location. Final disposition to the community for NPACER was 71 % (*n* = 172 of 243) compared with 60 % (116 of 194) people in crisis for the non-NPACER response (*p* = 0.02).

## Discussion

Over an eight-month period (2009–10), Victoria Police was directly involved in the mandatory transportation of 2401 people in some form of mental health crisis [13]. The clear majority of these people were transported directly to EDs. Given the strong indication of the negative experience of people in mental health crisis processed through EDs [5, 8, 17], alternative management pathways are required.

The major finding of this study was the ability of a combined police and mental health clinician second responder team to prevent the mandatory transportation of people in mental health crisis to the ED (see Figs. 2 and 3). This diversion was primarily to the least restrictive alternative environment, which involved assessment in the community and subsequent referral to a variety of community mental health and social care agencies (including community mental health centres, alcohol and other drug services, general practitioners, and accommodation options). Furthermore, a combined police and mental health clinician response better facilitated direct access to mental health inpatient services for those in mental health crisis, compared with direct access with a stand-alone police response in the state of Victoria [13].

The high rate of ED diversion to the community at initial contact in the current study contrasts to earlier findings where a second responder team resulted in 19 % of people in crisis remaining in the community at the point of initial assessment [14]. This disparity may be attributable to several factors, primary of which may be the substantial time difference between the two studies, locations (metropolitan Melbourne and Los Angeles) and the high rate of past criminal involvement of the cohort in the Lamb et al. [14] study.

Despite these benefits, a minority of people in mental health crisis in the current study were transported to a police station for assessment (see Fig. 3). Presumably, transportation to the police station was for safety reasons initiated by the front line first responder police. A further presumption is that this experience was transitory as most people were transferred to acute inpatient service or returned to their communities following assessment by NPACER. However, this study tells us little about the experience of people transported to police stations or the appropriateness of this diversion. Such diversion should be averted given the potential criminalization of those who are mentally ill through exposure to the criminal justice system [14, 18, 19]. Anecdotally, transportation to a police station in the current study was attributed to geographic efficiency so that the first responder police and NPACER could meet in a timelier manner. However, further investigation is required to thoroughly describe the pragmatic, circumstantial, and procedural processes that may explain this diversion to a potentially restrictive environment and if such diversion required subsequent presentation to an ED.

### Limitations

This study relied on retrospective data collected for reporting purposes and may be susceptible to selection bias as duplicate cases across the period were treated as independent episodes. Furthermore, the cross-sectional comparisons were time limited with no indication of the impact of the model on longitudinal outcomes such as the length of stay for those who entered into acute mental health inpatient services or on crisis relapse. Limitations in the research design give little insight into the economic implications of the model, sustainability of the model or its applicability to other area mental health services or jurisdictions. Furthermore, stakeholder (clinicians, police, people in crisis, and their families) perceptions of the benefits and limitations of the model are required to fully evaluate its impact. Further research which addresses these limitations is required.

## Conclusions

The selection and implementation of an acute mental health crisis response team is based on decisions related to the context of service delivery, the geographical scope of the divisional law enforcement area, resource constraints, collaboration among hospital EDs and other emergency services [20]. In the metropolitan growth corridor outlined in this study, a second responder team comprising a senior mental health clinician and a police officer enabled reductions in presentations to the ED by diverting people in mental health crisis to more appropriate and less restrictive environments. The model also facilitated direct admission to acute inpatient mental health services when people were assessed in the community, or transport to a police station for assessment. However, the appropriateness of the use of police stations to achieve these ends requires further investigation.

## Abbreviations

AMHS: Area Mental Health Service
ED: Emergency Department
NPACER: Northern Police and Clinician Emergency Response
